# Detection Performance Analysis of Array Dielectric Dispersion Logging Based on Sensitivity Function

**DOI:** 10.3390/s23125737

**Published:** 2023-06-20

**Authors:** Lianyun Cai, Shaogui Deng, Xiyong Yuan

**Affiliations:** 1 Key Laboratory of Deep Oil and Gas, China University of Petroleum (East China), Qingdao 266580, China; b18010047@s.upc.edu.cn (L.C.); upc_yxy@163.com (X.Y.); 2 Sinopec Matrix Corporation, Qingdao 266075, China

**Keywords:** array dielectric logging, detection characteristics, sensitivity function, dielectric constant, borehole geophysics

## Abstract

Dielectric logging is a critical method for exploring and developing complex oil and gas reservoirs, such as tight reservoirs, low-resistivity contrast reservoirs, and shale oil and gas reservoirs. The sensitivity function is extended to high-frequency dielectric logging in this paper. The detection characteristics of attenuation and phase shift of an array dielectric logging tool in different modes are investigated, along with the influencing factors such as resistivity and dielectric constant. The results show the following: (1) The symmetrical coil system structure makes the sensitivity distribution symmetrically distributed, and the detection range is more focused. In the same measurement mode, the depth of investigation (DOI) becomes deeper under high resistivity formation, and the sensitivity range oscillates outward when the dielectric constant becomes greater. (2) The DOIs of different frequencies and source spacings cover the radial zone between 1 cm and 15 cm. The detection range has been enlarged to include part of the invasion zones, improving the measurement data’s dependability. (3) With the increase in the dielectric constant, the curve tends to oscillate, and this behavior makes the DOI slightly shallower. Additionally, this oscillation phenomenon is obvious when the frequency, resistivity, and dielectric constant increase, particularly in high-frequency detection mode (F2, F3).

## 1. Introduction

Dielectric logging is a significant logging technique for distinguishing between oil and water. This logging method can directly measure the water saturation of the formation, and it is especially critical in tight gas sands, where water saturation can be very low and impossible to predict using standard resistivity logs. It also provides information about rock lithology, water porosity, and clay content [1,2,3,4,5,6,7]. The majority of the early dielectric logging methods use centered mandrel measurement. The receiving coils’ amplitude attenuation and phase shift reflect the change in complex permittivity [8], and the general working frequency is dozens of MHz, making the dielectric sensitivity insufficient. The Deep Electromagnetic Propagation Tool (DPT) from Schlumberger operates at a frequency of 25 MHz. The transmitting coils are equipped with a pair of near-receiving coils and a pair of far-receiving coils. The Electromagnetic Propagation Tool (EPT), introduced in 1977, embeds the transmitting and receiving antennas into the groove of the pad and measures the formation information by pushing the pad against the borehole wall. The operating frequency is increased to 1.1 GHz [9]. The High-Frequency Dielectric Logging Tool (HFD) was developed by Halliburton [10,11]. This push-type measurement method is less affected by the borehole under ideal push-type conditions. However, this kind of tool has some drawbacks, including a narrow detection range, loose pushing when the borehole is irregular, difficulty eliminating high-frequency measurement errors, and insufficient information from single-frequency measurements. Atlas introduced dual-frequency (47, 200 MHz) dielectric logging equipment after the 1980s. The 47 MHz mode uses a centered measurement approach, whereas the 200 MHz mode uses a pad placed against the borehole wall for measurement, allowing for both deep and shallow detection depths. At the same time, the flushing zone and invasion zone information is measured [12,13].

According to the development history, dielectric logging tools have been developed in multi-frequency and multi-source spacing logging modes. The Array Dielectric Logging Tool (ADT) from Schlumberger uses multi-frequency and multi-source spacing detection modes to measure formation information, overcoming the limitations of traditional dielectric logging’s shallow detection depth and insufficient measurement information [14,15,16,17,18]. The Array Dielectric Logging Tool has been successfully used in the evaluation of reservoirs with fresh water and heavy oils, and because of its high vertical resolution it has been used for the evaluation of laminated reservoirs [19,20]. With the development of unconventional oil and gas resources, the application of array multi-frequency dielectric logging tools in oil fields is growing, both at home and abroad. Studying the detection sensitivity of array dielectric logging is therefore of great theoretical and practical importance [3,21,22,23,24,25].

The sensitivity function (also known as the geometric factor or response function) has been used to characterize the spatial distribution characteristics of logging responses for the formation of electrical parameters, which can be useful for analyzing instrument detection performance and data processing [26,27]. The Doll geometric factor was first developed to describe the geometric distribution of the independent unit ring’s contribution distribution to the induction logging response [28]. Gianzero, Moran, Howard, and Zhang extended the geometric factor theory’s applicability to the condition of low-resistivity formation as the frequency increases [29,30,31,32]. For the calculation of the sensitivity function, McGillivray proposed the difference approximation and the Fréchet differential method [33]. Tølbøll then investigated the detection sensitivity of different dipole systems based on the Fréchet derivatives [34]. Wang et al. extended the Born geometric factor to the anisotropic formation and simulated the spatial distribution characteristics of geometric factors of multicomponent induction logging [35,36]. The operating frequency of electromagnetic logging is generally high, and resistivity is calibrated using attenuation and phase shift. Based on the geometric factor theory, Zhou et al. investigated the phase-shift response function of the EPRL instrument [37]. Xing et al. extended the Born approximation theory of electromagnetic fields to derive the response function of attenuation and phase shift to dielectric constant and conductivity, but without providing a method to quantitatively identify the detection range [38]. Deng et al. defined a sensitivity function for electromagnetic logging while drilling based on difference approximation theory, and they quantitatively described the detection ranges of different frequencies under different resistivity conditions [39]. Zhang et al. studied the attenuation and phase-shift sensitivity of logging while drilling extra-deep azimuthal resistivity measurements based on the extended Born theory [40].

However, current research on sensitivity function is generally based on induction logging or electromagnetic logging-while-drilling responses. The following problems still exist when it is applied to array dielectric logging: (a) Electromagnetic waves decay rapidly due to the strong skin effect at high frequencies, and the dielectric logging response is heavily influenced by the formation parameters; the applicability of the sensitivity function is not clear under these conditions. (b) The influence of the dielectric constant is always neglected in current research, but it is not to be ignored, as the frequency increases in array dielectric logging. (c) There are a lot of data that need to be processed in array dielectric logging curves, and it is necessary to figure out the sensitivity of each detection mode to determine the priority of the inversion parameters. In a word, finding a novel and proper method that can explain the detection characteristics of high-frequency electromagnetic logging tools is important, and clarifying the detection performance of different modes is of significance for the inversion and data processing of the array dielectric tool.

To solve the above problems, this paper extends the electromagnetic sensitivity function to array dielectric logging. It obtains a set of theories to represent the spatial distribution characteristics of attenuation and phase shift. First, the sensitivity function expression of the basic coil system is derived, and the spatial distribution characteristics of its sensitivity function in different detection modes are simulated and analyzed in a homogeneous medium under different electrical parameters. Simultaneously, the depth of investigation is quantitatively characterized by a radial integral sensitivity function, and its applicability is investigated. Finally, the vertical resolution of the array dielectric logging is investigated using various layer thickness models.

## 2. Method

**Figure 1:**
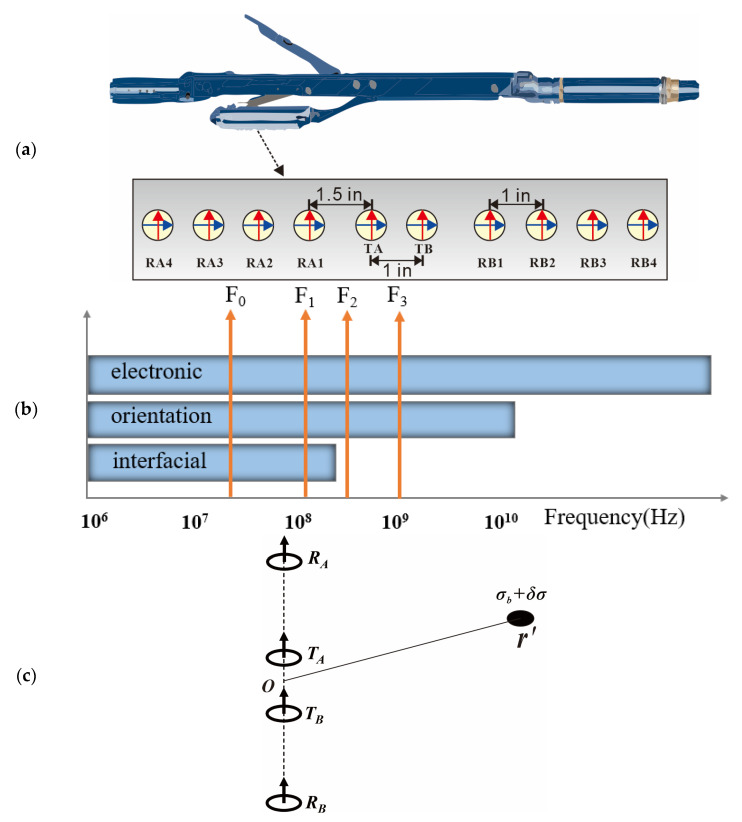
Operating frequencies and pad coil configurations of the Array Dielectric Logging Tool: (**a**) Two transmitters (TA, TB) and eight receivers (RA1-4, RB1-4) are symmetrically distributed in a push arm; the spacing between adjacent transmitters (receivers) is 1 inch, and the spacing between adjacent transmitters and receivers is 1.5 inches. (**b**) Operation frequencies of the tool; 20 MHz, 200 MHz, 500 MHz, and 1 GHz (F0-F3) are chosen to cover the entire interfacial polarization spectrum of dielectric dispersion. (**c**) The basic symmetrical coil system in homogeneous formation with an abnormal body; the attenuation and phase shift definitions are based on this symmetrical four-coil system; the midpoint *O* is defined as the origin; the complex conductivity of the background formation is *σ*; a small abnormal body with complex conductivity *σ+δσ* and volume *V_s_* is assumed at the point of ***r*′**.

As shown in Figure 1b, the array dielectric logging tool selects four operating frequencies covering the frequencies of different polarization mechanisms. F0 = 20 M, F1 = 200 M, F2 = 500 M, and F3 = 1 GHz are the specific frequencies. Additionally, symmetrically distributed two-transmitter and eight-receiver coils are employed for measurement. The spacing between each transmitting coil is approximately 1 inch (0.0254 cm), and the spacing between the TA and RA1, TB, and RB1 is 1.5 inches (0.0381 cm). Each transmitting and receiving coil set contains horizontal and vertical coils that can be taken as magnetic dipoles. When the instrument is turned on, the voltage signals of various *xx* and *zz* components are measured. For the *zz* component, each set of transmitting and receiving coils can be simplified to the structural combination shown in Figure 1c, and all frequency and source spacing (*L*) combinations are shown in Table 1. For example, F0L0 means a four-coil system operated at 20 MHz and with source spacing of [−2, −0.5, 0.5, 2] inches.

To investigate the electromagnetic propagation characteristics and detection sensitivity to the surrounding area of various dielectric scanner detection modes, as shown in Figure 1c, we set the location of the receiver at ***r_T_*** and the receiver at ***r***, and the coil was regarded as a magnetic dipole. When there is an abnormal body in the formation, the complex conductivity can be equivalent to ***σ*** = ***σ****_b_ + δ**σ***, ***σ*** = σ* − *iω**ε***, where ***σ****_b_* is the background conductivity, and *δ**σ*** is the change in the abnormal body conductivity compared with the background medium. The electric field changes caused by the abnormal body can be written as follows [33,34,38]:

(1) $$\delta E\left(r\right)=E\left(r\right)-E_{p}\left(r\right)=i\omega \mu_{0}\int_{V_{s}}dr^{\prime }\delta \sigma \left(r^{\prime }\right)G\left(r,r^{\prime }\right)\cdot E\left(r^{\prime }\right)$$

***E****_p_* is the primary field when the dipole source is located at a homogeneous background medium. ***E***(***r*′**) is the electric field inside the abnormal body; it can be replaced by the background field ***E****_p_*(***r*′**) based on Born approximation. ***G***(***r***,***r*′**) is a green function contributed to a receiving signal from an abnormal body; it can be calculated by placing a dipole source at ***r*′** and computing the response at ***r***. The expression of the primary field and green function in the homogeneous formation can be derived analytically [35]. The voltage in the receivers can be expressed as follows:

(2) $$V\left(r\right)=\oint_{L}E\left(r\right)dl=2\pi rE\left(r\right)=\left|V\right|e^{i\theta }$$

The combination of Equation (1) and the definition of attenuation and phase shift based on a single-transmitter double-receiver coil system can be written as follows:

(3) $$\frac{V_{A}^{}\left(r_{1}\right)}{V_{B}^{}\left(r_{2}\right)}=\frac{\left|V_{A}^{}\right|e^{i\theta_{1}}}{\left|V_{B}^{}\right|e^{i\theta_{2}}}=Ae^{i\Delta \theta }$$

where *A* and Δ*θ* represent the attenuation and phase shift, respectively. *V_A_*, *V_B_* can represent any two receiving signals. The geometric factor of electromagnetic logging can be obtained by deriving the logarithm of the above formula.

(4) $$S_{1}=\delta \ln A+i\delta \Delta \theta =\frac{\delta V_{R_{A}T_{A}}^{}}{V_{R_{A}T_{A}}^{}}-\frac{\delta V_{R_{A}T_{B}}^{}}{V_{R_{A}T_{B}}^{}}$$

For the symmetrical coil system shown in Figure 1c, the sensitivity function of array dielectric logging can be expressed as follows:

(5) S=12δVRATAVRATA−δVRATBVRATB−δVRBTBVRBTB+δVRBTAVRBTASAtt=Re(S)SPS=Im(S)

To analyze the depth of investigation (DOI) of each measurement mode of array dielectric logging, the radial integral sensitivity function can be obtained by integrating the sensitivity function *S* of dielectric logging. Under this situation, the abnormal body (*V_s_*) is integrated into an abnormal zone that extends vertically and indefinitely; the formation becomes a radially symmetric two-layered model; the complex conductivity of the inner layer is *σ + δσ,* while that of the outer layer is *σ*; *S_HI_* corresponds to the radius of the interface between two layers (*r*), and it is normalized between [0, 1] when *r* changes from 0 to ∞; the DOI is the corresponding *r* when *S_HI_* meets the average line.

(6) $$S_{HI}=\int_{-\infty }^{\infty }\int_{0}^{r}Sdrdz$$

**Figure 2:**
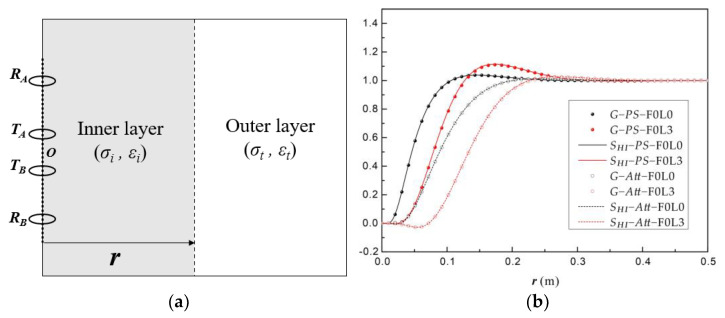
Radial integrated sensitivity function *S_HI_* compared with pseudo−geometric factor *G*; *R* = 1 Ω·m, *ε_r_* = 1: (**a**) Radially symmetric two-layered model used for the calculation of the pseudo−geometric factor *G*; the array dielectric tool is simplified to a basic four−coil system. (**b**) *S_HI_* and *G* change with *r* (corresponding to the maximum point of the integration for *S_HI_* and the radius of the inner layer for *G*), including *Att* and *PS* under the F0L0 and F0L3 detection modes.

To verify this algorithm, we established a cylindrical two-layer model, as shown in Figure 2a, including the inner layer (*σ_i_, ε_i_*) and the outer layer (*σ_t_, ε_t_*). A pseudo-geometric factor *G* was defined due to the responses during the change in radial depth ***r***, as shown in Equation (7), where *f* is the response of dielectric logging (*Att* or *PS*), *f*(*σ_i_, ε_i_*) and *f*(*σ_t_, ε_t_*) represent the response in a homogeneous formation with (*σ_i_, ε_i_*) and (*σ_t_, ε_t_*), respectively, and *f*(*σ_t_, ε_t;_ r, σ_i_, ε_i_*) is the response when both layers exist. The electromagnetic field of a magnetic dipole located in a cylindrical two-layer formation can be calculated by a pseudo-analytical solution based on a general transmission and reflection matrix [23,41].

**Figure 3:**
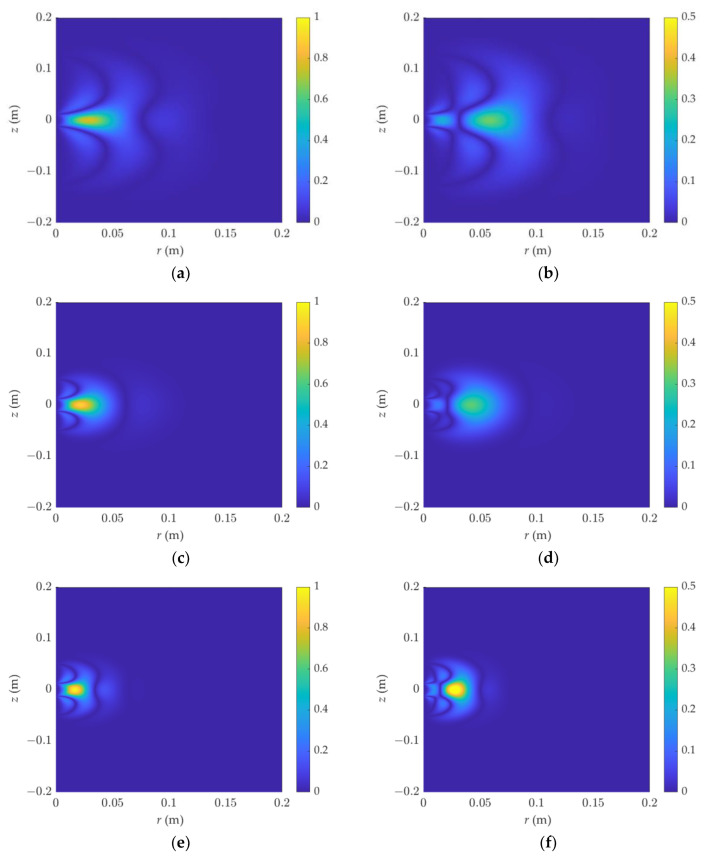
The 2D sensitivity function varies with frequency and source spacing. R = 1 Ω·m, and *ε_r_* = 1. (**a**) PS, F1L3, representing the phase shift 2D sensitivity function when F1 = 200 MHz, L3 = [−5, −0.5, 0.5, 5] inch. The same goes for the following; the detection modes are illustrated in Table 1: (**b**) Att, F1L3, (**c**) PS, F1L0, (**d**) Att, F1L0, (**e**) PS, F3L0, (**f**) Att, F3L0.

Next, we calculated the results of the Array Dielectric Logging Tool by both the radial integrated sensitivity function *S_HI_* and the pseudo-geometric factor *G*, as shown in Figure 3b. As for the radial integrated sensitivity function *S_HI_*, we set *R* = 1 Ω·m, *ε_r_* = 1; meanwhile, as for the pseudo-geometric factor *G*, (*σ_t_, ε_t_*) = (1, 1), and (*σ_i_, ε_i_*) must be close to (*σ_t_, ε_t_*), where a minor difference of 1% is imposed that (*σ_i_, ε_i_*) = (1 + 1%)(*σ_t_, ε_t_*), because only when the electrical difference between the two layers is small enough are the results of *S_HI_* and *G* theoretically equivalent. As shown in Figure 3b, the results of *S_HI_* (solid lines for phase shift and dashed lines for attenuation) and *G* (solid cycles for phase shift and hollow cycles for attenuation) were consistent with one another in different detection modes; thus, the effectiveness of the algorithm was validated.

(7) $$\frac{1-G}{f(\sigma_{i},\varepsilon_{i})}+\frac{G}{f(\sigma_{t},\varepsilon_{t})}=\frac{f(\sigma_{t},\varepsilon_{t};r,\sigma_{i},\varepsilon_{i})}{f(\sigma_{i},\varepsilon_{i})\cdot f(\sigma_{t},\varepsilon_{t})}$$

## 3. Results and Discussion

Figuring out the relative contribution of each formation part around the borehole to the logging response is important for the accurate evaluation of the formation parameters. The Array Dielectric Logging Tool has many detection modes with different frequencies and source spacings; the detection characteristics of each mode are simulated and discussed in this section, including the two-dimensional sensitivity, depth of investigation, and vertical resolution. This might be helpful for the processing of array dielectric logging data. 

### 3.1. Two-Dimensional Sensitivity Distribution

Based on the formation model shown in Figure 1c, we simulated the sensitivity functions when the location ***r*′** of the abnormal body changed; since the tool was simplified as a coil array placed along the *z*-axis, the electromagnetic fields ***E****_p_*(***r*′**) and ***G***(***r***,***r*′**) in Equation (1) were rotationally symmetrical, leading to the rotational symmetry of the sensitivity function. Thus, we only present the results in the *rOz* plane. The two-dimensional sensitivity distribution of attenuation and phase shift under different detection modes are modeled below.

Set *R* = 1 Ω·m, *ε_r_* = 1. Figure 3 depicts the results. The sensitivity distribution is symmetric in the higher and lower ranges, and it diffuses in the radial direction. The sensitive area is within 10 cm in this case. The detection range widens radially and the detection depth is deeper in the long-source-spacing mode. When the frequency is high, the sensitivity function is more concentrated than in F3L0, and the detection depth is shallow but the sensitivity is high. At the same time, when the left and right maps are compared, the amplitude distribution is more dispersed, the detection range is larger than the phase shift, and it is highly affected by the surrounding strata. The phase shift’s sensitive area is centered at the well, and the sensitivity ranges change slightly longitudinally, with a good detection resolution.

**Figure 4:**
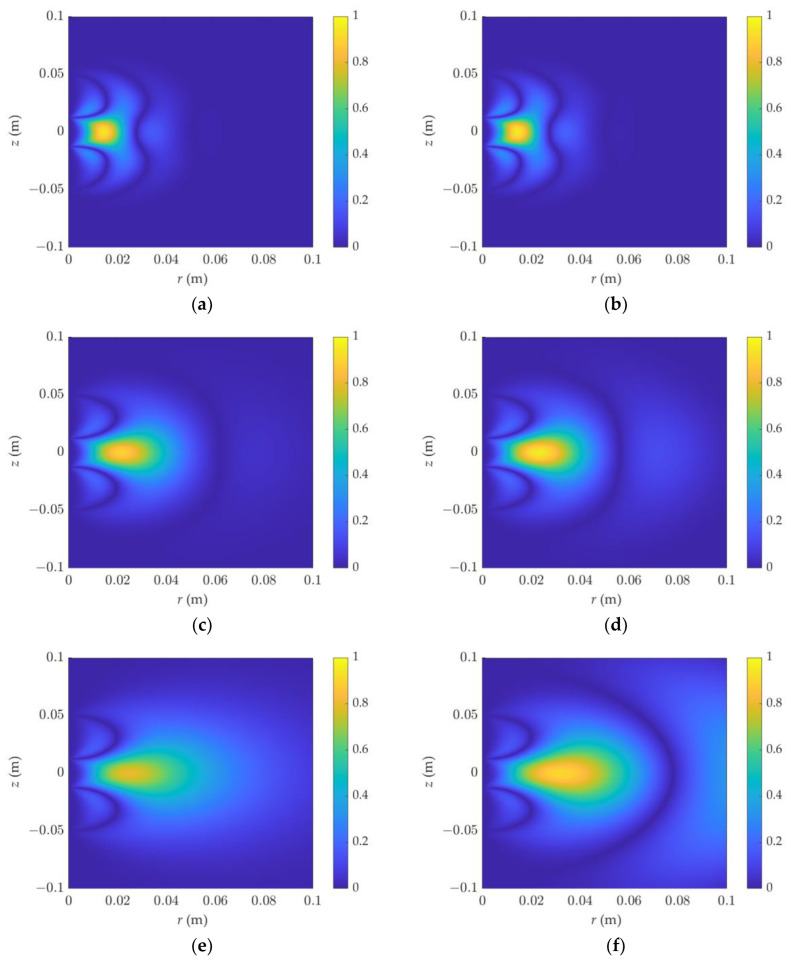
The 2D phase-shift sensitivity function of dielectric logging varies with resistivity and dielectric constant; the detection mode is F1L0: (**a**) *ε_r_* = 1, R = 0.1 Ω·m, (**b**) *ε_r_* = 40, R = 0.1 Ω·m, (**c**) *ε_r_* = 1, R = 1 Ω·m, (**d**) *ε_r_* = 40, R = 1 Ω·m, (**e**) *ε_r_* = 1, R = 10 Ω·m, (**f**) *ε_r_* = 40, R = 10 Ω·m.

By varying the formation resistivity and dielectric constant, the distribution of the phase shift sensitivity function in the F1L0 detection mode was investigated. As shown in Figure 4, the relative dielectric constant on the left is 1, while that on the right is 40, and the resistivity from top to bottom is 0.1, 1, and 100 Ω·m, respectively. In Figure 4a,b, they look similar with different dielectric constants when the resistivity is 0.1 Ω·m. It can be seen that under low-resistivity conditions, the dielectric constant has little effect on the phase shift. When the resistivity increases, the detection range increases significantly. At the same time, under high-resistivity conditions, when the dielectric constant is large, the detection range of the phase-shift signal oscillates slightly and diffuses outward.

**Figure 5:**
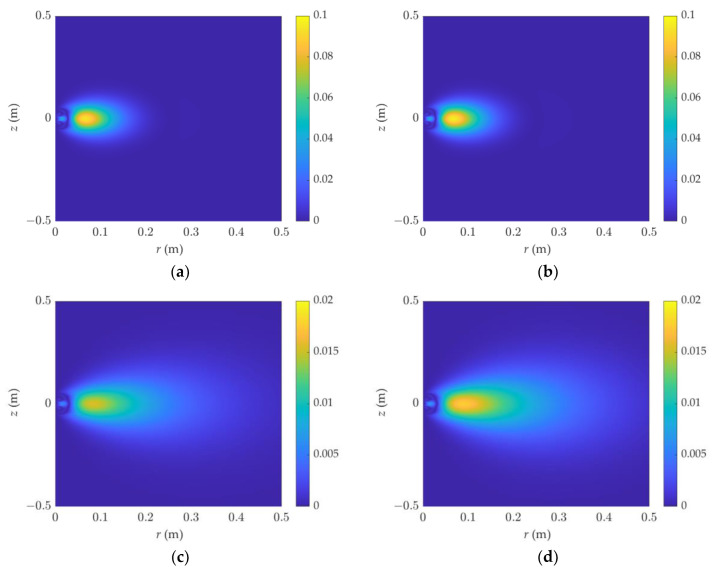
The 2D attenuation sensitivity function of dielectric logging varies with resistivity and dielectric constant: (**a**) *ε_r_* = 1, R = 1 Ω·m, (**b**) *ε_r_* = 40, R = 1 Ω·m, (**c**) *ε_r_* = 1, R = 10 Ω·m, (**d**) *ε_r_* = 40, R = 10 Ω·m. The detection mode is F0L0.

**Figure 6:**
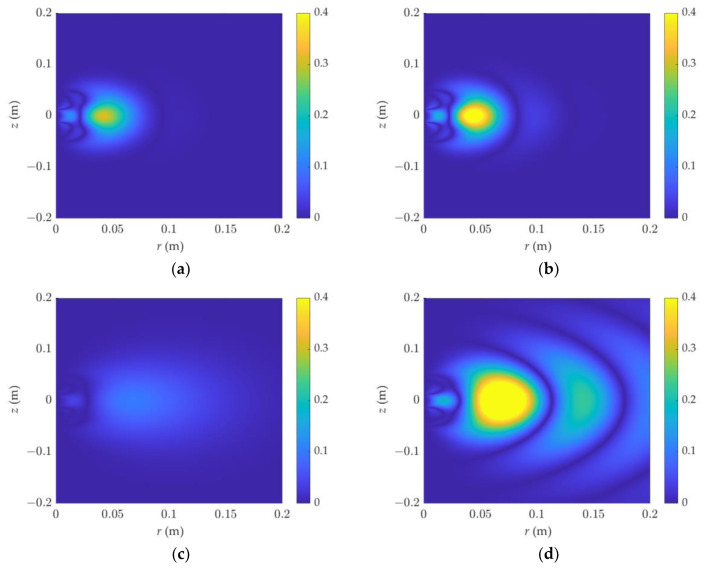
The 2D attenuation sensitivity function of dielectric logging varies with resistivity and dielectric constant: (**a**) *ε_r_* = 1, R = 1 Ω·m, (**b**) *ε_r_* = 40, R = 1 Ω·m, (**c**) *ε_r_* = 1, R = 10 Ω·m, (**d**) *ε_r_* = 40, R = 10 Ω·m. The detection mode is F1L0.

At the same time, the distribution characteristics of the attenuation sensitivity in the F0L0 and F1L0 detection modes were simulated, as shown in Figure 5 and Figure 6, respectively. Because of the huge shift in the sensitivity magnitude, the color interval in the graph is not uniform. Figure 6 demonstrates that when the resistivity and the dielectric constant increase, the attenuation sensitivity zone diffuses outward, with the diffusion being most visible at a high dielectric constant. Figure 6 shows the attenuation detection sensitivity in F1L0 mode. The dielectric constant increases with frequency, generating apparent rhythmic fluctuations, and the sensitivity distribution is not concentrated.

**Figure 7:**
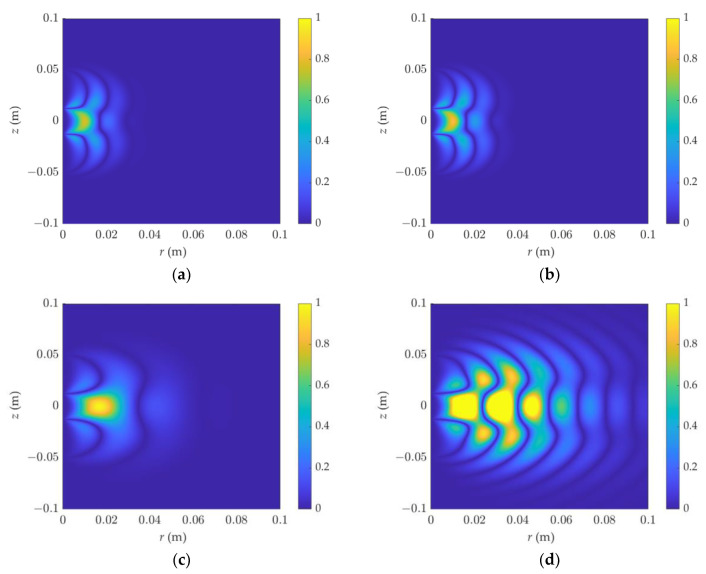
The 2D phase-shift sensitivity function of dielectric logging varies with resistivity and dielectric constant: (**a**) *ε_r_* = 1, R = 0.1 Ω·m, (**b**) *ε_r_* = 40, R = 0.1 Ω·m, (**c**) *ε_r_* = 1, R = 1 Ω·m, (**d**) *ε_r_* = 40, R = 1 Ω·m. The detection mode is F3L0.

**Figure 8:**
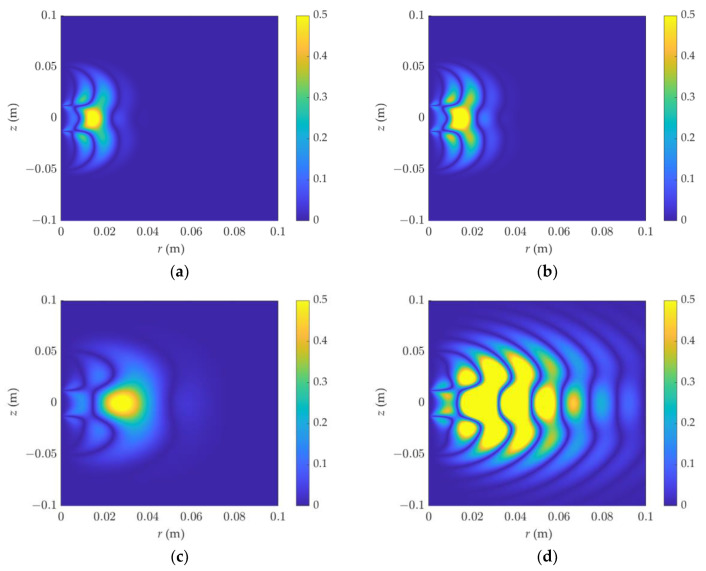
The 2D attenuation sensitivity function of dielectric logging varies with resistivity and dielectric constant: (**a**) *ε_r_* = 1, R = 0.1 Ω·m, (**b**) *ε_r_* = 40, R = 0.1 Ω·m, (**c**) *ε_r_* = 1, R = 1 Ω·m, (**d**) *ε_r_* = 40, R = 1 Ω·m. The detection mode is F3L0.

As illustrated in Figure 7 and Figure 8, the sensitive areas of phase shift and attenuation in the F3L0 detection mode were simulated. Under high-frequency and low-resistivity conditions, the phase-shift signal is affected by the dielectric constant. When the resistivity increases, the dielectric constant has a greater influence, and the signal oscillates strongly. It is not appropriate at this time to describe the detection range of dielectric logging by the sensitivity function.

### 3.2. Depth of Investigation (DOI)

**Figure 9:**
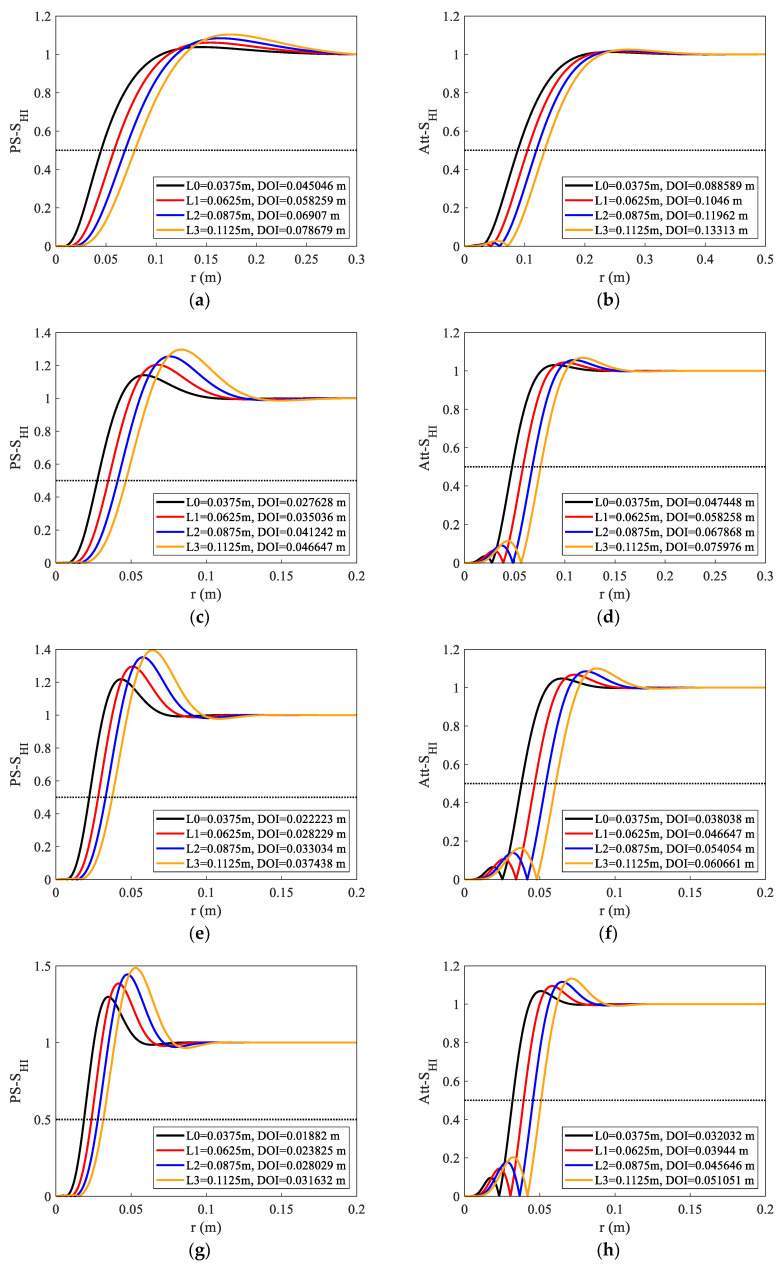
Phase shift and attenuation radial integrated sensitivity functions under different frequencies (F0~F3) and source spacings (L0~L3); the legend of each panel shows the DOIs from L0~L3. The frequencies of each panel are as follows: (**a**) F0, Ps, (**b**) F0, Att, (**c**) F1, Ps, (**d**) F1, Att, (**e**) F2, Ps, (**f**) F2, Att, (**g**) F3, Ps, (**h**) F3, Att.

Firstly, the influences of DOI at different frequencies and source spacings were simulated. The resistivity was set to 1 Ω·m and the relative dielectric constant was 1. The abscissa in Figure 9 represents the radial depth, and the ordinate represents the radial integral sensitivity function. The figure’s four curves correspond to the four source spacings. The curve value is the absolute value and tends to be 1 as the radial depth increases. At the frequency F0 = 20 MHz, the phase-shift DOI was between 0.045 and 0.078 m, and the attenuation DOI was between 0.088 and 0.133 m, as determined by the analysis. The DOI increased linearly with the increase in the source spacing, and the DOI of the amplitude ratio was between 1.5 and 2 times the phase shift. The phase shift DOI was 0.018~0.031 m and the attenuation DOI was 0.032~0.051 m for F3 = 1 GHz. The DOI becomes shallower with the increase in the frequency, and the curve corner becomes obvious. The DOIs of different frequencies and source spacings cover the radial zone between 2 cm and 13 cm under this formation condition. Additionally, some detection modes share the same DOI, making the data more reliable and robust to characterize the formation accurately.

**Figure 10:**
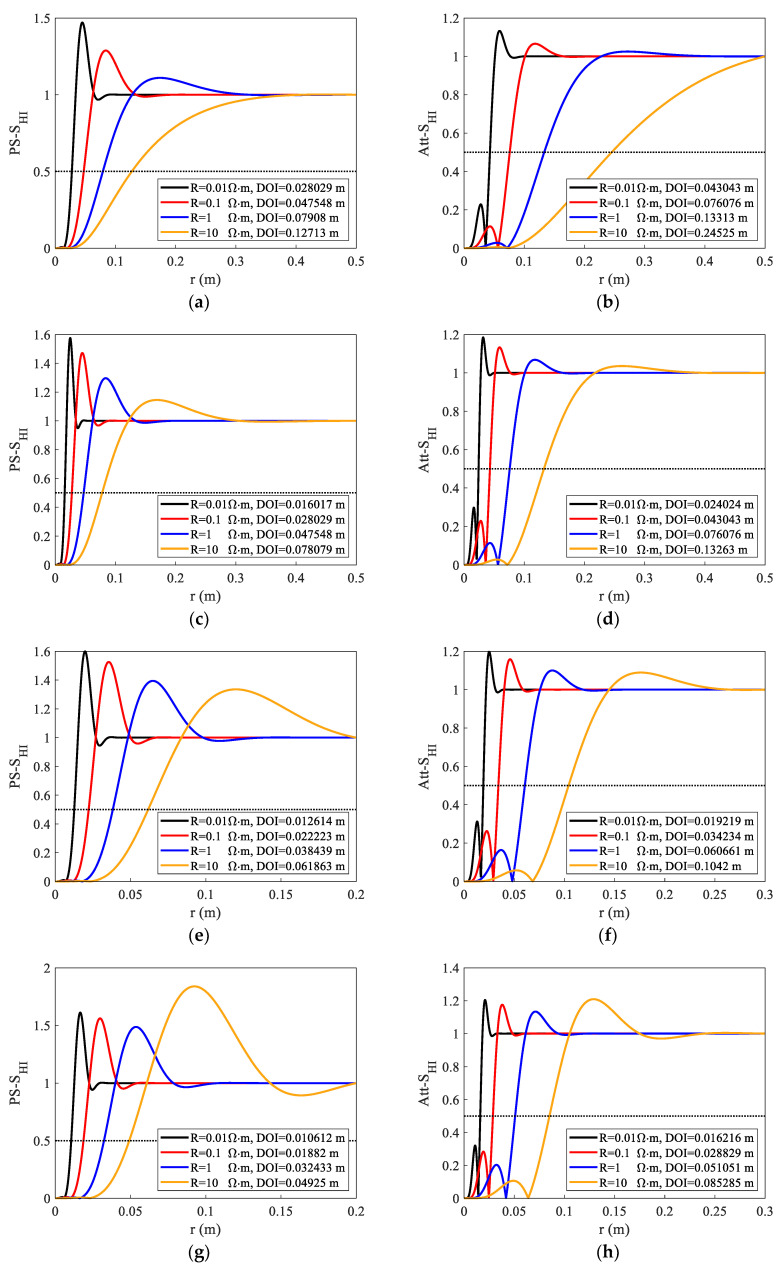
Phase shift and attenuation radial integrated sensitivity functions under different resistivity, *ε_r_* = 1. The legend of each figure shows the DOIs when the resistivity changes from 0.01 Ω·m to 10 Ω·m. The detection modes of each figure are as follows: (**a**) F0L3, Ps, (**b**) F0L3, Att, (**c**) F1L3, Ps, (**d**) F1L3, Att, (**e**) F2L3, Ps, (**f**) F2L3, Att, (**g**) F3L3, Ps (**h**) F3L3, Att.

The effect of varying resistivity on the DOI was simulated, as shown in Figure 10. We set resistivity changes from 0.01 Ω·m to 10 Ω·m; the relative dielectric constant was 1, and we selected L3 as the source spacing and simulated the radial integral sensitivity functions at four frequencies. Figure 10 shows that the DOI changes significantly with the change in resistivity. In Figure 10a, the *Att* DOI changes from 4.3 cm to 24.5 cm when the resistivity changes from 0.01 Ω·m to 10 Ω·m. In Figure 10h, the *Att* DOI can still reach 8.5 cm at high frequency (F3 = 1 GHz) when the resistivity is 10 Ω·m. However, the DOI of most of the detection modes ranges from 1 to 10 cm. This outperforms typical high-frequency dielectric logging. It is no longer confined to measuring mud cakes and flushing zones around the well, and the detection range is enlarged to include some invasion zones, improving the measurement data’s dependability.

**Figure 11:**
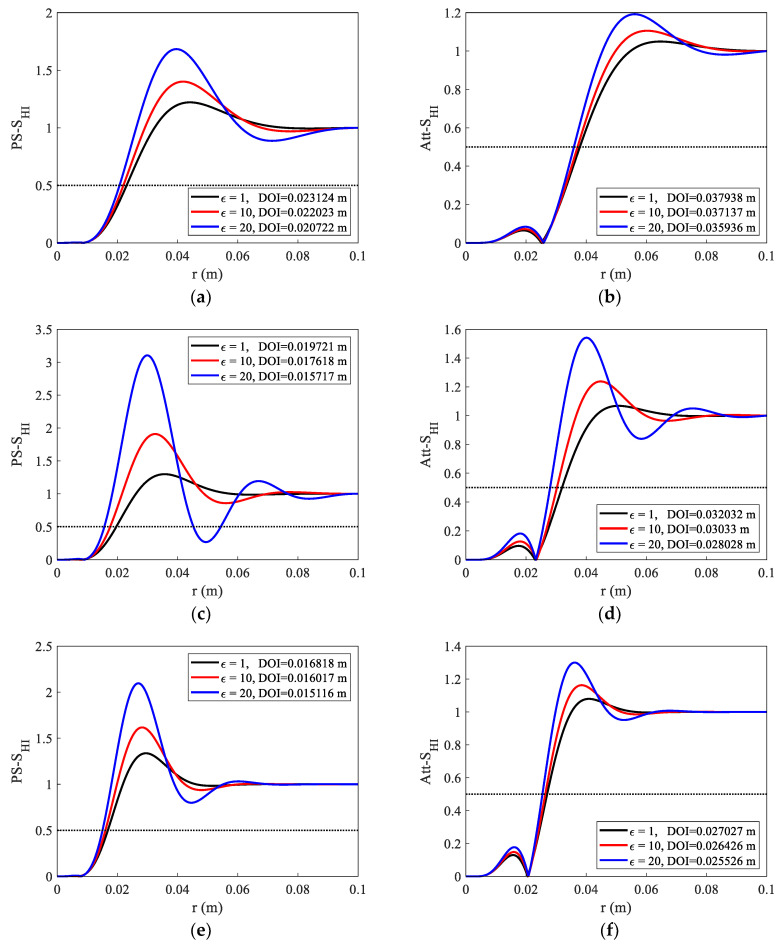
Phase shift and attenuation radial integrated sensitivity functions under different dielectric constants; two resistivity values were set; the legend of each panel shows the DOIs when the relative dielectric constant changes from 1 to 20; the detection modes and parameters of each figure are as follows: (**a**) F2L0, Ps, R = 1 Ω·m, (**b**) F2L0, Att, R = 1 Ω·m, (**c**) F3L0, Ps, R = 1 Ω·m, (**d**) F3L0, Att, R = 1 Ω·m, (**e**) F3L0, Ps, R = 0.5 Ω·m, (**f**) F3L0, Att, R = 0.5 Ω·m.

We set the relative dielectric constant changes from 1 to 20. The influence of different dielectric constants on the DOI was studied under detection modes F2L0 and F3L0. Additionally, two resistivity values (0.5 Ω·m, 1 Ω·m) were chosen to be simulated. Figure 11a,b show that the PS curve changes more than the Att curve when the dielectric constant changes from 1 to 20; comparing them with panels (c) and (d), the dielectric constant has a greater influence on the DOI at a higher frequency. With the increase in the dielectric constant, the curve seems to oscillate, and this behavior makes the DOI slightly shallower. When the dielectric constant and resistivity continue to rise, the curve’s oscillation becomes so intense that it is impossible to quantify its detection properties, particularly in high-frequency detection mode (F2, F3). 

### 3.3. Vertical Resolution

**Figure 12:**
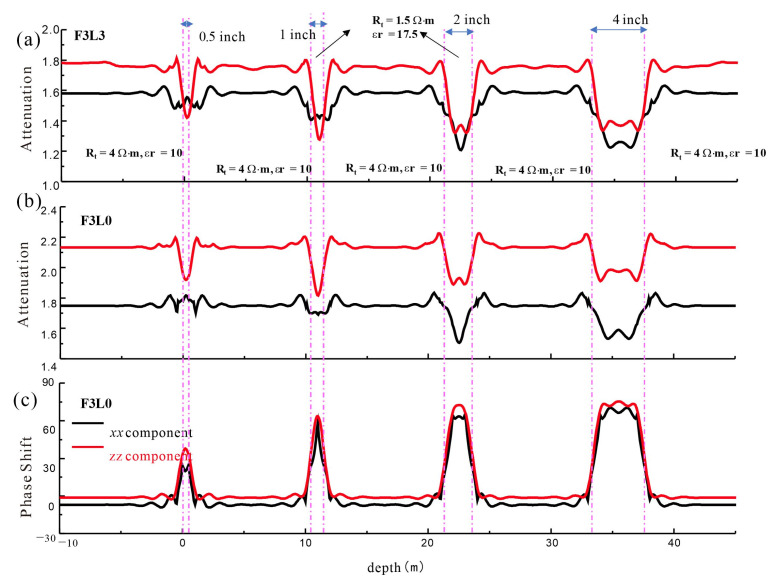
The vertical resolution of high-frequency detection mode of array dielectric logging. The red line indicates the *zz* component and the black line indicates the *xx* component. (**a**) Attenuation curve of detection mode F3L3. (**b**) Attenuation curve of detection mode F3L0. (**c**) Phase shift of detection mode F3L0.

A formation model with various layer thicknesses was built to investigate the vertical resolution of array dielectric logging, and the phase-shift and attenuation curves of various detection modes were simulated. As shown in Figure 12, the resistivity of the surrounding rock was 4 Ω·m, the relative dielectric constant was 10, the resistivity of the middle interlayer was 1.5 Ω·m, and the relative dielectric constant was 17.5. The interlayers were constructed with thicknesses of 0.5, 1, 2, and 4 inches. The red line is the *zz* component curve, and the black line is the *xx* component curve. The figure shows that the *zz* component attenuation curve can distinguish a 1-inch thin layer, that the formation thickness determined by the half-=amplitude point is more accurate, and that the curve amplitude is close to the true value of formation resistivity, whereas the *xx* component amplitude deviates from the true value when 0.5 and 1 inch are used. The phase shift curve can better depict the thin-layer information. In summary, the coaxial component *zz* outperforms the coplanar component *xx*, and the attenuation and phase-shift signals can better provide the formation conductivity and dielectric constant information. Compared with the phase shift, the attenuation is more susceptible to the surrounding rock. Near the formation contact, the coplanar component is substantially nonlinear, and the larger the source spacing, the stronger the nonlinearity. When the longitudinal resolution of dielectric logging is combined with the spatial geometric factor distribution under different detection modes, it is clear that the vertical resolution is related to the distance between the two transmitting coils, and the longitudinal resolution of dielectric logging can reach 1 inch.

## 4. Conclusions

This paper investigated the relative contribution of dielectric constant and resistivity for array dielectric logging responses based on sensitivity function, with the following findings:

(1) The DOIs of different frequencies and source spacings cover the radial zone between 1 cm and 15 cm. This outperforms typical high-frequency dielectric logging. It is no longer confined to measuring mud cakes and flushing zones around the well, and the detection range is enlarged to include some invasion zones, improving the measurement data’s dependability.
(2) With the increase in the dielectric constant, the curve tends to oscillate, and this behavior makes the DOI slightly shallower. When the dielectric constant and resistivity continue to rise, the curve’s oscillation becomes so intense that it is impossible to quantify its detection properties, particularly in high-frequency detection mode (F2, F3).
(3) The vertical resolution is related to the spacing between the two transmitting coils and can reach 1 inch, which proves to be able to evaluate the laminated formation.

The detection performance analysis based on several detection modes has an impact on the inversion and data processing of the array dielectric logging measurements. The high-sensitivity properties of different detection modes can be studied based on the detection range to reduce the amount of calculation.

## Figures and Tables

**Table 1:** Detection modes of the Array Dielectric Logging Tool.

	F (Hz)	F0 = 20 M	F1 = 200 M	F2 = 500 M	F3 = 1 G
L (Inch)					
L0 = [−2, −0.5, 0.5, 2]		F0L0	F1L0	F2L0	F3L0
L1 = [−3, −0.5, 0.5, 3]		F0L1	F1L1	F2L1	F3L1
L2 = [−4, −0.5, 0.5, 4]		F0L2	F1L2	F2L2	F3L2
L3 = [−5, −0.5, 0.5, 5]		F0L3	F1L3	F2L3	F3L3

## Data Availability

Not applicable.
